# Supplementary material for: Non-Invasive Prediction of IDH Mutation in Patients with Glioma WHO II/III/IV Based on F-18-FET PET-Guided In Vivo 1H-Magnetic Resonance Spectroscopy and Machine Learning
Source: Cancers (Basel). 2020 Nov 17;12(11):3406. doi: 10.3390/cancers12113406 (PMC7698334; doi:10.3390/cancers12113406)
Supplement: Supplementary file 1 [file cancers-12-03406-s001.zip › Cancers_983274_Supplement.pdf]

Supplementary material: Non-invasive prediction of *IDH* mutation in patients with glioma WHO II/III/IV based on F-18-FET PET-guided *in vivo* ^{1}H-magnetic resonance spectroscopy and machine learning

Elisabeth Bumes[1*#], Fro-Philip Wirtz[2#], Claudia Fellner[3], Jirka Grosse[4], Dirk Hellwig[4], Peter J. Oefner[2], Martina Häckl[2], Ralf Linker[1], Martin Proescholdt[5], Nils Ole Schmidt[5], Markus J. Riemenschneider[6], Claudia Samol[2], Katharina Rosengarth[5], Christina Wendl[3], Peter Hau[1], Wolfram Gronwald[2+], Markus Hutterer[1,6+]

[1]Department of Neurology and Wilhelm Sander-NeuroOncology Unit, Regensburg University Hospital, Regensburg, Germany

[2]Institute of Functional Genomics, University of Regensburg, Regensburg, Germany

[3]Department of Radiology and Division of Neuroradiology, Regensburg University Hospital, Regensburg, Germany

[4]Department of Nuclear Medicine, Regensburg University Hospital, Regensburg, Germany

[5]Department of Neurosurgery, Regensburg University Hospital, Regensburg, Germany

[6]Department of Neuropathology, Regensburg University Hospital, Regensburg, Germany

[7]Department of Neurology, Saint John of God Hospital Linz, Linz, Austria

[#]shared first authorship, [+]shared last authorship

*Correspondence: elisabeth.bumes@ukr.de; Tel.: +0049-0941-944-18751

Supplementary Material: Figure S1

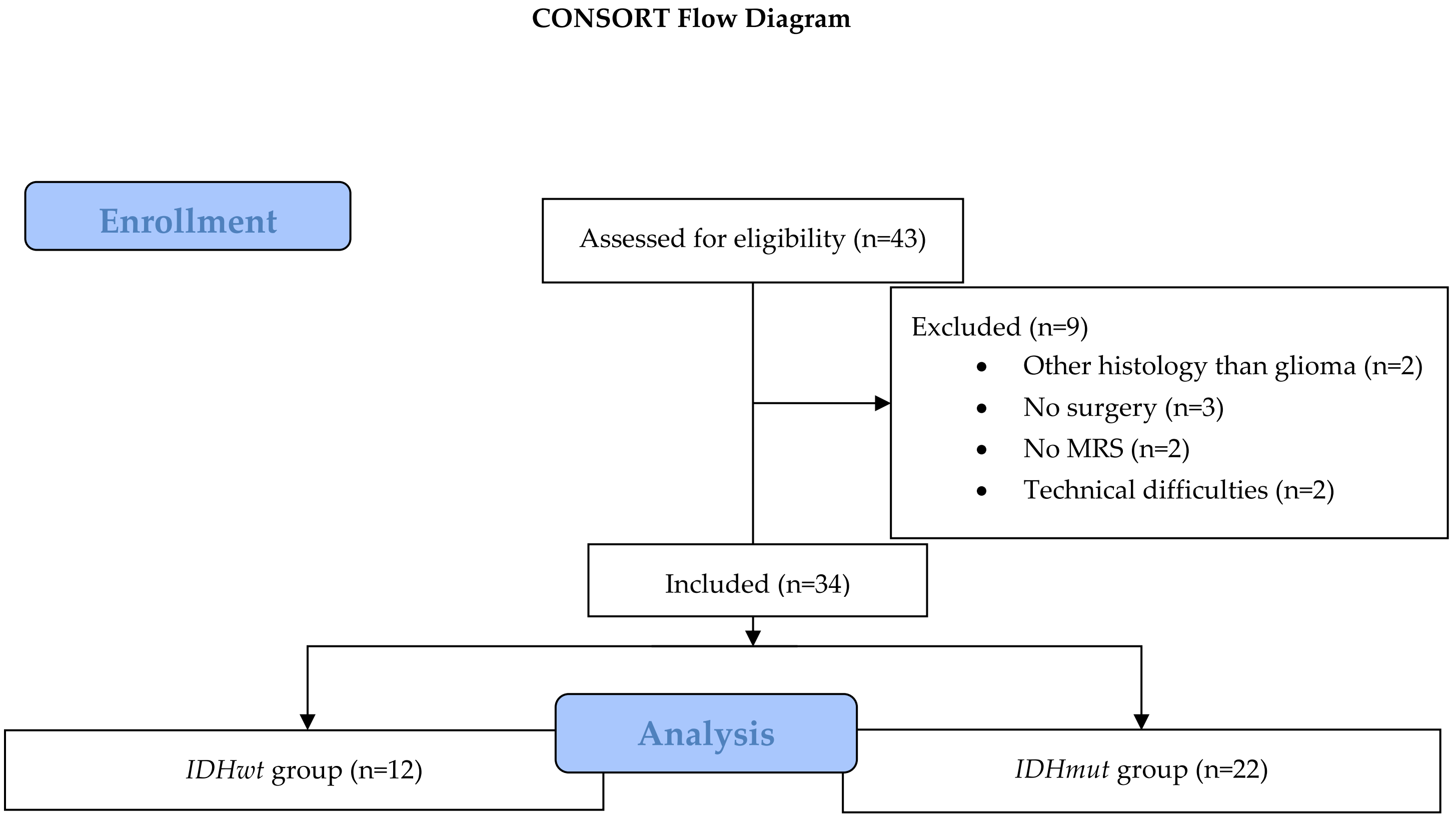

Figure S1: Consort flow diagram showing the patient enrollment.

Supplementary Material: Figure S2

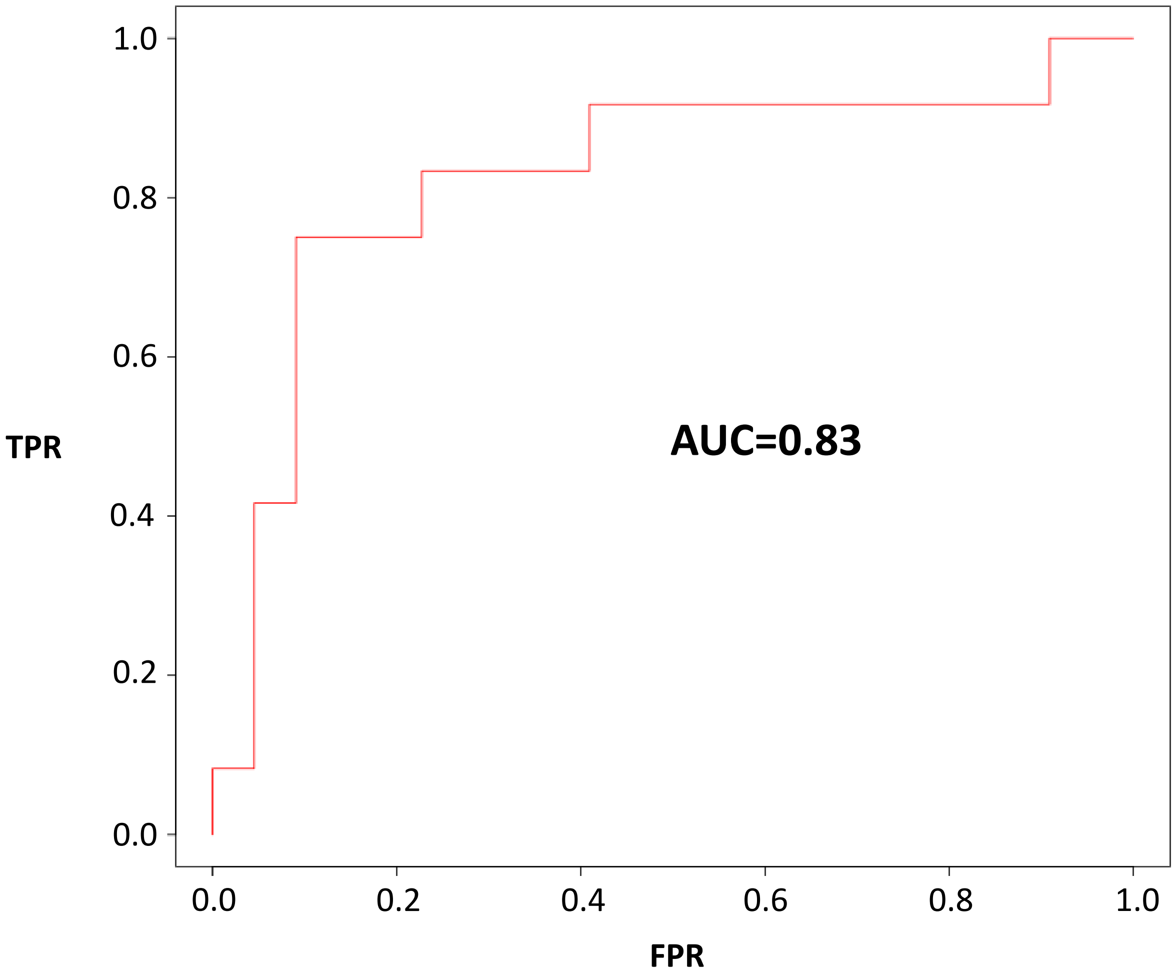

Figure S2. Prognostication of IDH status by linear SVM. Prognostication performance was assessed by analysis of the ROC curve.
